# The Law Relating to Medical Officers of Health (Outside|Metropolis).—IV

**Published:** 1903-07-18

**Authors:** J. E. R. Stephens

**Affiliations:** Barrister-at-Law, etc.


### THE LAW RELATING TO MEDICAL OFFICERS OF HEALTH (OUTSIDE METROPOLIS).-IV.

By J. E. R. Stephens, Barrister-at-Law, etc.

(*Continued from Page* 251.)

*Buildings Unfit for Human Habitation.*—Under section 30 of the Housing of the Working Classes Act, 1890, it is the duty of the medical officer of health of every district to represent to the local authority of that district any dwelling-house which appears to him to be in a state so dangerous or injurious to health as to be unfit for human habitation. The representation made by the medical officer of health must be in writing. The premises must be in a state dangerous or injurious to health. Mere want of repair would not necessarily fulfil this condition.

By section 31 of the above Act, if in any district any four or more householders living in or near to any street complain in writing to the medical officer of health of that district that any dwelling-house in or near that street is in a condition so dangerous or injurious to health as to be unfit for human habitation, he shall forthwith inspect the same, and transmit to the local authority the said complaint, together with his opinion thereon, and if he is of opinion that the dwelling-house is in a condition dangerous or injurious to health, shall represent the same to the local authority, but the absence of any such complaint shall not excuse him from inspecting any dwelling-house and making a representation thereon to the local authority. If within three months after receiving the said complaint and opinion or representation of the medical officer, the local authority, not being in the administrative county of London, or not being a rural sanitary authority in any other county, declines or neglects to take any proceedings to put this part of this Act in force, the householders who signed such complaint may petition the Local Government Board for an inquiry, and the said board, after causing an inquiry to be held, may order the local authority to proceed under this part of this Act.

*Disinfection of Bedding.*—Section 6 of The Infectious Disease (Prevention) Act, 1890, provides that any local authority, or the medical officer of health of any local authority in that behalf, may by notice in writing require the owner of any bedding, clothing, or other articles which have been exposed to the infection of any infectious disease to cause the same to be delivered over to an officer of the local authority for removal for the purpose of disinfection; and any person who fails to comply with such a requirement shall be liable to a penalty not exceeding £10. The bedding, clothing, and articles shall be disinfected by the authority, and shall be brought back and delivered to the owner free of charge, and if any of them suffer any unnecessary damage, the authority shall compensate the owner for the same, and the amount of compensation shall be recoverable in, and in case of dispute shall be settled by, a court of summary jurisdiction.

The local authority are not to be liable for all damage which may be done by the removal and disinfection of the bedding, etc., but only for such damage as may have been unnecessary, i.e., such as might with reasonable care have been avoided. The above section does not interfere with the power of the local authority to direct the destruction of the bedding, etc., on payment of compensation under section 121 of the Public Health Act, 1875.

*Inspection of Dairies.*—It is enacted by section 4 of the Infectious Disease (Prevention) Act, 1890, that in case the medical officer of health is in possession of evidence that any person in the district is suffering from infectious disease attributable to milk supplied within the district from any dairy, situate within or without the district, or that the consumption of milk from such dairy is likely to cause infectious disease to any person residing in the district, such medical officer shall, if authorised in that behalf by an order of a justice having jurisdiction in the place where such dairy is situate, have power to inspect such dairy, and if accompanied by a veterinary inspector or some other properly qualified veterinary surgeon to inspect the animals therein, and if on such inspection the medical officer of health shall be of opinion that infectious disease is caused from consumption of the milk supplied therefrom, he shall report thereon to the local authority, and his report shall be accompanied by any report furnished to him by the said veterinary inspector or veterinary surgeon, and the local authority may thereupon give notice to the dairyman to appear before them within such time, not less than twenty-four hours, as may be specified in the notice, to show cause why an order should not be made requiring him not to supply any milk therefrom within the district until such order has been withdrawn by the local authority, and, if in the opinion of the local authority, he fails to show such cause, then the local authority may make such order; and the local authority shall forthwith give notice of the facts to the sanitary authority and county council (if any) of the district or county in which such dairy is situate, and also to the Local Government Board. Any person refusing to permit the medical officer of health on the production of such order to inspect any dairy, or if so accompanied as aforesaid to inspect the animals kept there, or after any, such order not to supply milk as aforesaid has been given supplying any milk within the district in contravention of such order, or selling it for consumption therein, shall be deemed guilty of an offence against this Act.

*Inspection of Meat and Other Foods.*—Any medical officer of health or inspector of nuisances may, under section 116 of the Public Health Act, 1875, at all reasonable times inspect and examine any animal, carcase, meat, poultry, game, flesh, fish, fruit, vegetables, corn, bread, flour, or milk, exposed for sale, or deposited in any place for the purpose of sale, or of preparation for sale, and intended for the food of man, the proof that the same was not exposed or deposited for any such purpose, or was not intended for the food of man, resting with the party charged; and if any such animal, carcase, meat, poultry, game, flesh, fish, fruit, vegetables, corn, bread, flour, or milk appears to such medical officer or inspector to be diseased or unsound, or unwholesome or unfit for the food of man, he may seize and carry away the same himself or by an assistant, in order to have the same dealt with by a justice.

The words in the above section are specific, and do not include every kind of food. The bye-laws of municipal corporations sometimes provide for the seizure of unwholesome food; as in *Shillito* v. *Thompson* (39 J. P. 773) a defendant was held to be rightly convicted under such bye-law for selling unwholesome cheese. But where the local authority have adopted the Public Health Acts Amendment Act, 1890, section 289, that Act enacts that sections 116–119 of the Public Health Act, 1875 (relating to unsound meat), shall extend and apply to all articles intended for the food of man, sold, or exposed for sale, or deposited in any place for the purpose of sale, or of preparation for sale within the district of any local authority. A justice may condemn any such article, and order it to be destroyed or disposed of, as mentioned in Section 117 of the Act of 1875, if satisfied on complaint being made to him that such article is diseased, unsound, unwholesome, or unfit for the food of man, although the same has not been seized, as mentioned in Section 116 of the Act of 1875. The object of the enactment seems to be to meet cases where the seizure of the meat has not been in all respects in conformity with the requirements of Section 116. For example, in *Vinter* v. *Hind* (47 J.P. 373) a butcher exposed for sale part of a cow which had died of disease, and sold the meat to a customer, who took it home for food and some days afterwards at the request of the appellant, an inspector of nuisances, handed it over to him, and it was condemned by a justice as unfit for the food of man. It was held that this was not seized and condemned in manner provided by Section 116, and that the butcher could not be convicted. Section 118 of the Act of 1875 enacts that any person who in any manner prevents any medical officer of health or inspector of nuisances from entering any premises and inspecting any animal, carcase, meat, poultry, game, flesh, fish, fruit, vegetables, corn, bread, flour or milk exposed or deposited for the purpose of sale, or of preparation for sale, and intended for the food of man, or who obstructs or impedes any such medical officer or inspector, or his assistant, when carrying into execution the provisions of this Act, shall be liable to a penalty not exceeding £5.

On complaint made on oath by a medical officer of health or by an inspector of nuisances, or other officer of a local authority, any justice may grant a warrant to any such officer to enter any building, or part of a building, in which such officer has reason for believing that there is kept or concealed any animal carcase, meat, etc., which is intended for sale for the food of man, and is diseased, unsound or unwholesome, or unfit for the food of man ; and to search for, seize, and carry away any such animal or other article in order to have the same dealt with by a justice under the provisions of this Act. Any person who obstructs any such officer in the performance of his duty under such warrant shall, in addition to any other punishment to which he may be subject, be liable to a penalty not exceeding £20 (section 119). If an officer suspects concealment he cannot enter forcibly until he obtains a warrant under this section.

*Common Lodging-houses.*—Under section 84 of the Public Health Act, 1875, the keeper of a common lodging-house shall, when a person in such house is ill of fever or any infectious disease, give immediate notice thereof to the medical officer of health of the local authority, and also to the Poor Law relieving officer of the union or parish in which the common lodging-house is situated. And under section 85, the keeper of a common lodging-house, and every other person having, or acting in the care or management thereof, shall, at all times when required by any officer of the local authority, give him free access to such house or any part thereof, and any such keeper or person who refuses such access shall be liable to a penalty not exceeding £5. This section does not require such officer to produce any specific authority for his demand of inspection.

*Notification of Infectious Disease.*—By section 3 of the Infectious Diseases (Notification) Act, 1889, where an inmate of any building used for human habitation, is suffering from an infectious disease to which this Act applies, then, unless such building is a hospital in which persons suffering from an infectious disease are received, the following provisions shall have effect:—

(*a*) The head of the family to which such inmate (in this Act referred to as the patient) belongs, and in his default the nearest relatives of the patient present in the building, or being in attendance on the patient, and in default of such relatives every person in charge of or in attendance on the patient, and in default of any such person the occupier of the building shall, as soon as he becomes aware that the patient is suffering from an infectious disease to which this Act applies, send notice thereof to the medical officer of health of the district:

(*b*) Every medical practitioner attending on or called in to visit the patient shall forthwith, on becoming aware that the patient is suffering from an infectious disease to which this Act applies, send to the medical officer of health for the district a certificate stating the name of the patient, the situation of the building, and the infectious disease from which, in the opinion of such medical practitioner, the patient is suffering. Every person required by this section to give a notice or certificate who fails to give the same shall be liable, on summary conviction, to a fine of 40s. Provided that if a person is not required to give notice in the first instance, but only in default of some other person, he shall not be liable to any fine if he satisfies the Court that he had reasonable cause to suppose that the notice had been duly given. The local authority shall pay to every medical practitioner for each certificate duly sent by him in accordance with this Act a fee of 2s. 6d. if the case occurs in his private practice, and of 1s. if the case occurs in his practice as medical officer of any public body or institution.

*Obstructive Buildings.*—Section 38 of the Housing of the Working Classes Act, 1890, enacts that if a medical officer of health finds that any building within his district, although not in itself unfit for human habitation, is so situate that by reason of its proximity to or contact with any other buildings, it causes one of the following effects, that is to say:—

(*a*) It stops ventilation, or otherwise makes or conduces to make such other buildings to be in a condition unfit for human habitation or dangerous or injurious to health; or

(*b*) It prevents proper measures from being carried into effect for remedying any nuisance injurious to health or other evils complained of in respect of such other buildings; in any such case the medical officer of health shall represent to the local authority the particulars relating to such first-mentioned building (in this Act referred to as "an obstructive building") stating that in his opinion it is expedient that the obstructive building should be pulled down.

*Unhealthy Areas.*—Section 4 of the Housing of the Working Classes Act 1890 provides that where an official representation is made to the local authority that within a certain area in the district of such authority, either—

(*a*) Any houses, courts, or alleys are unfit for human habitation, or

(*b*) The narrowness, closeness, and bad arrangement, or the bad condition of the streets and houses, or groups of houses within such area, or the want of light, air, ventilation, or proper conveniences, or any other sanitary defects, or one or more of such causes, are dangerous or injurious to the health of the inhabitants either of the buildings in the said area or of the neighbouring buildings; and that the evils connected with such houses, etc., cannot be effectually remedied otherwise than by an improvement scheme for the re-arrangement and reconstruction of the streets and houses within such area, or of some of such streets or houses, the local authority shall take such representation into their consideration. An official representation for the purposes of this part of this Act means a representation made to the local authority by the medical officer of health.

---

THE cost of the new wing of the Walthamstow Hospital, recently opened by the Duke and Duchess of Connaught, is some £6,000. The new wing comprises a ward for 14 patients, new out-patients' department, mortuary and operating and casualty rooms.

